# Endoplasmic Reticulum (ER) Stress and Its Role in Pancreatic β-Cell Dysfunction and Senescence in Type 2 Diabetes

**DOI:** 10.3390/ijms23094843

**Published:** 2022-04-27

**Authors:** Ji-Hye Lee, Jaemin Lee

**Affiliations:** 1 Department of New Biology, Daegu Gyeongbuk Institute of Science and Technology (DGIST), Daegu 42988, Korea; idea0723@dgist.ac.kr; 2 New Biology Research Center, Daegu Gyeongbuk Institute of Science and Technology (DGIST), Daegu 42988, Korea; 3 Well Aging Research Center, Daegu Gyeongbuk Institute of Science and Technology (DGIST), Daegu 42988, Korea

**Keywords:** endoplasmic reticulum, ER stress, pancreatic beta cell, cellular senescence, type 2 diabetes, insulin, islet amyloid polypeptide

## Abstract

An increased life span and accompanying nutritional affluency have led to a rapid increase in diseases associated with aging, such as obesity and type 2 diabetes, imposing a tremendous economic and health burden on society. Pancreatic β-cells are crucial for controlling glucose homeostasis by properly producing and secreting the glucose-lowering hormone insulin, and the dysfunction of β-cells determines the outcomes for both type 1 and type 2 diabetes. As the native structure of insulin is formed within the endoplasmic reticulum (ER), ER homeostasis should be appropriately maintained to allow for the proper metabolic homeostasis and functioning of β-cells. Recent studies have found that cellular senescence is critically linked with cellular stresses, including ER stress, oxidative stress, and mitochondrial stress. These studies implied that β-cell senescence is caused by ER stress and other cellular stresses and contributes to β-cells’ dysfunction and the impairment of glucose homeostasis. This review documents and discusses the current understanding of cellular senescence, β-cell function, ER stress, its associated signaling mechanism (unfolded protein response), and the effect of ER stress on β-cell senescence and dysfunction.

## 1. Introduction

As the world globally becomes an aging and affluent society, aging-associated metabolic disorders such as obesity and type 2 diabetes prevail rapidly and become a serious health burden. Aging is a natural process in which the functions of body tissues deteriorate over time. It is regarded as a fatal regression that all living organisms will experience. As organisms age, the accumulation of senescent cells eventually leads to damage at the level of the body tissue. Senescent cells not only cause dysfunction in the body tissue but also trigger inflammation, the formation of cancer cells, and senescence in neighboring cells. Therefore, preventing or slowing senescence would effectively ameliorate age-related physiological disorders and systematic inflammation [1].

Among the various factors that influence aging, impaired protein homeostasis (proteostasis) has been found to significantly induce aging and age-related diseases. The production and function of chaperones decline with age, as do the autophagy-lysosome and ubiquitin-proteasome systems. All of these are crucial for maintaining adequate proteostasis; thus, their decline in function leads to dysfunction in the protein quality control system, an accumulation of unfolded or misfolded proteins, and the development of a range of aging-related illnesses [2,3].

For example, type 2 diabetes is a disease associated with both obesity and aging. Senescent cell accumulation in the organs involved in the process of metabolism either cause or are caused by obesity and diabetes [4]. Thus, aging is one of the primary risk factors for the development of type 2 diabetes and other metabolic disorders [4]. Type 2 diabetes is initiated by peripheral insulin resistance but eventually progresses to the dysfunction and loss of β-cells, which are a critical group of pancreatic cells that regulate glucose homeostasis by producing and secreting insulin in response to blood glucose level changes. The level of pancreatic β-cell functioning also declines with age, according to several recent investigations in animals and humans [5,6,7,8,9].

Insulin is initially translated as proinsulin, and its native structure is formed in the endoplasmic reticulum (ER). It is further processed in the secretory vesicles, where it eventually becomes bioactive insulin [10]. The ER is a membrane–bound organelle and is widely distributed throughout the cytoplasm of pancreatic β-cells. It is responsible for the folding and quality control of the secretory and transmembrane proteins that are transported to the appropriate locations after proper structure formation and modification occur. As insulin folding includes the formation of proper disulfide bonds crucial for insulin’s biological function and takes place mainly in the ER, maintaining proper ER proteostasis is critical. Indeed, numerous studies have suggested that ER dysfunction exacerbates both type 1 and type 2 diabetes [11,12,13]. Moreover, ER dysfunction results in ER stress that accompanies other cellular stresses, such as oxidative stress, inflammation, and mitochondrial stress, all of which crucially affect β-cell dysfunction and the development of metabolic and aging-associated illnesses.

This paper aims to review the current understanding of β-cell biology and its dysfunction during aging and the progression of type 2 diabetes. In particular, we focused on ER stress and its critical role in insulin synthesis and secretion, along with the β-cell dysfunction that occurs during the progression of type 2 diabetes and aging.

## 2. Pancreatic β-Cell Biology and Its Dysfunction in Aging and Type 2 Diabetes

### 2.1. Insulin Secretion and Biosynthesis in Pancreatic β-Cells

**Figure 1:**
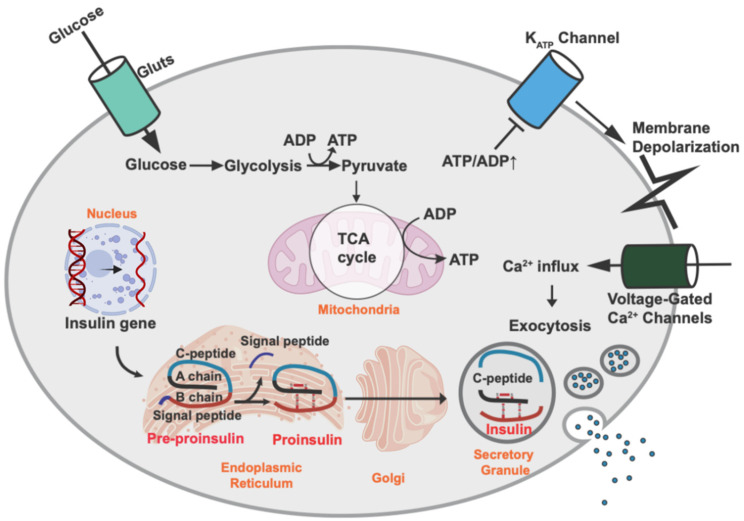
Insulin biosynthesis and secretion by pancreatic β-cells. Insulin biosynthesis begins with transcription of the insulin gene in the nucleus and translation of the mRNA into pre-proinsulin. Upon translocation into the ER, the signaling peptide is removed, thereby creating proinsulin. Proinsulin is folded and stabilized by a disulfide bond in the ER and then transported into the secretory granules, where proinsulin is cleaved to C-peptide and insulin. Glucose is the major stimulus of insulin secretion. Glucose is transported across the β-cell plasma membrane via the glucose transporter [GLUT2 (rodent) or GLUT1 (human)]. Then, glucose is metabolized to pyruvate via glycolysis and further metabolized and produces ATP in the mitochondria through oxidative phosphorylation. An increase in the ATP/ADP ratio leads to closure of the ATP-sensitive K^+^ channels and membrane depolarization, which results in the opening of L-type voltage-gated Ca^2+^ channels (L-VGCCs). The influx of extracellular calcium ions into the β cells induces the exocytosis of secretory vesicles with insulin.

Pancreatic β-cells are unique endocrine cells that synthesize, store, and secrete insulin and are assembled into rosette-like structures around blood vessels. They are coupled by gap junctions to allow them to respond to changes in blood sugar levels, facilitate the uptake of other types of fuel, and release appropriate amounts of insulin into the blood circulation [14]. The process of glucose-stimulated insulin secretion (GSIS) via changes in intracellular adenosine triphosphate (ATP) levels is well documented (Figure 1). When blood glucose levels are increased in a healthy body, glucose crosses the β-cell plasma membrane via the glucose transporter GLUT2 (rodent) or GLUT1 (human). Next, glucose is metabolized to pyruvate via glycolysis pathways and is then metabolized in the mitochondria through oxidative phosphorylation to produce ATP. The increase in the ATP/adenosine diphosphate (ADP) ratio leads to the closure of the ATP-sensitive K^+^ channels and the depolarization of the membrane potential, which subsequently opens L-type voltage-gated Ca^2+^ channels (L-VGCCs). Extracellular calcium ions enter the β-cells via L-VGCCs, which in turn causes the induction of exocytosis of secretory vesicles to secrete insulin [15].

Insulin that is lost from intracellular stores via stimulated insulin granule exocytosis is rapidly replenished by the upregulation of insulin biosynthesis in pancreatic β-cells in a healthy individual [10]. The transcription of the insulin gene is regulated primarily by transcription factors, such as Pdx1, NeuroD1, MafA, and Pax6. These transcription factors are also crucially involved in pancreatic development, β-cell maturation, and survival [16,17,18].

The insulin gene encodes a 110-amino acid precursor called pre-proinsulin, which contains a signal peptide, an A chain, a B chain, and a C-peptide. Insulin is a posttranslationally processed product of insulin precursors (Figure 1). Pre-proinsulin is translocated into the ER with the help of a signal recognition particle and becomes proinsulin after its signal peptide has been removed. Proinsulin folds rapidly in the ER lumen once the signal peptide has been removed. Human insulin consists of 21 amino acid residues in the A chain (A1–21) and 30 amino acid residues in the B chain (B1–30). Proinsulin also contains six cysteines that form three disulfide bonds: two disulfide bonds between the A and B chains (A7–B7, A20–B19) and one intra-A chain disulfide bond (A6–A11) [19]. Insulin (proinsulin) folding in the ER is prompted by enzymes that catalyze the formation of proper disulfide bonds and the breakage of any inappropriate bonds because insulin must have the proper disulfide bonds to form its native structure and have viable efficacy [20,21,22]. The oxidizing condition is crucial in the formation of disulfide bonds, and protein disulfide isomerase (PDI) plays an important role as an ER redox chaperone by mediating both the reduction and oxidation of disulfide bonds [23,24,25]. Additionally, the ER oxidoreductin 1 (Ero1 in yeast and ERO1-L in mammals) is required for the oxidation of glutathione and the reduced state of proteins, such as PDI, in the ER [26,27]. Other ER-resident folding factors include the amino acid cis-trans isomerases, N-glycosylation enzymes, and chaperones such as GRP94, GRP78 [also called Ig heavy chain binding protein (BiP)], calnexin, and calreticulin [28,29]. In addition to assisting in protein folding, ER chaperones also retain proteins that are newly translated but have not yet fully formed their native structure in the ER. They will not do so until they are maturely folded [23]. The importance of proinsulin folding is well exemplified in the Akita mouse, one of the most well-known diabetic animal models, in which cysteine (the seventh amino acid of the insulin A chain) was substituted with tyrosine. The substitution of cysteine with tyrosine leads to the prevention of the formation of proper disulfide bonds between the A and B chains, resulting in proinsulin misfolding, subsequent ER stress, eventual β-cell failure, and diabetes [30]. Additionally, several human insulin mutations were found to induce insulin misfolding, ER stress, β-cell failure, and diabetes, similar to those of the Akita mouse model [31].

### 2.2. Calcium Signaling in β-Cells

Maintaining Ca^2+^ homeostasis is critical for proper β-cell function, and its impairment is strongly associated with type 2 diabetes progression. In the β-cells, elevated cytosolic Ca^2+^ concentration induces insulin granule exocytosis. Furthermore, the chronic stimulation of Ca^2+^ signaling has been shown to elevate ER stress and contribute to β-cells dysfunction [32]. Extracellular Ca^2+^ enters into β-cells primarily through L-VGCCs, inducing insulin secretion. Conversely, cytosolic Ca^2+^ is removed via plasma membrane Ca^2+^-ATPase (PMCA) and the Na^+^/Ca^2+^ exchanger (NCX) [33,34,35]. Cytosolic Ca^2+^ is also stored in the ER and mitochondria, which is mediated by the sarco-endoplasmic reticulum Ca^2+^-ATPases (SERCAs) for the ER and voltage-dependent anion-selective channel proteins (VDACs) and the mitochondrial Ca^2+^ uniporter (MCU) complex for the mitochondria [32,36]. Stored ER Ca^2+^ is released to the cytoplasm via two Ca^2+^ channels in the ER, inositol-1,4,5-triphosphate receptors (IP_3_Rs) and the ryanodine receptor (RyR) [37].

Apart from directly affecting insulin secretion via membrane potential changes, cytosolic Ca^2+^ also contributes to insulin production via Ca^2+^ signaling, which is mediated by calmodulin and its downstream signaling of the Ca^2+^/calmodulin-dependent protein kinase (CaMK)/cAMP response binding protein (CREB) and the calcineurin/nuclear factor of activated T cells (NFAT) [32].

Islets of diabetes mouse models exhibited poor calcium fluxes and Ca^2+^ oscillations in response to glucose stimulation [38,39]. Additionally, reduced PMCAs and VGCCs expression was observed in type 2 diabetes rodent islets [40,41]. Impaired Ca^2+^ oscillations in diabetic islets are partly due to the reduction of SERCA activity [39], and the depletion of ER Ca^2+^ levels by reduced SERCA action leads to ER stress and β-cell death [42].

### 2.3. Pancreatic β-Cell Proliferation

The expansion of the pancreatic β-cell mass is active from the embryonic stage to the neonatal period, whereas adult β-cell proliferation occurs only under limited conditions such as pregnancy and obesity-induced insulin resistance. The β-cell proliferation rate in human pancreatic islets is the highest in neonates (around 2% of β-cells exhibit Ki67 positivity) but drops to about 0.5% after childhood [43]. During the fetal period, β-cells are generated mainly from endocrine progenitor cells, whereas during the neonatal period, new β-cells are generated from existing β-cells. Whether β-cells remain in quiescence or under cell division is regulated by CDK and its inhibitors [44,45]. The progression of the cell cycle of β-cells is driven by the cyclin D–CDK4/6 complex, which is inhibited by CDK inhibitors, such as p16^Ink4a^, p21^Cip1^, and p27^Kip1^ [46]. The deletion of cyclin D2 (*Ccnd2*^−/−^) in β-cells fails to stimulate an adequate compensatory upregulation of cyclin D1 or D3. Thus, it drastically attenuates postnatal β-cell proliferation and mass, resulting in progressive glucose intolerance and eventual diabetes [47,48,49]. Likewise, in the study with mice, the forced expression of p27^Kip1^ in β-cells during embryonic and postnatal periods impaired their ability to proliferate, leading to a reduction in β-cell mass and the development of diabetes [50]. Conversely, the deletion of p27^Kip1^ (*Cdkn1b*^−/−^) increases the proliferation of β-cells under normal circumstances, as well as in genetic models of insulin resistance and diabetes (*Irs2*^−/−^ or *db/db*), the latter of which exhibits an improvement in glucose homeostasis [50,51]. Furthermore, the loss of the Rb protein in islet progenitors increases neurogenin 3 (Ngn3)-expressing precursors, with enhanced β-cell differentiation and neogenesis, whereas a deficiency in the Rb protein decreases differentiation in α-cells and increases their conversion to β-cells. A deficiency in the Rb proteins in both α- and β-cells leads to an induction of E2F1, but it results in opposing p53 levels (being increased in α-cells but decreased in β-cells), which leads to the further loss of postnatal α-cells and the expansion of functional β-cells via the induction of p53-mediated cell death [52].

### 2.4. Pancreatic β-Cell Dysfunction in Aging and Type 2 Diabetes

Aging is a major risk factor for type 2 diabetes, in which peripheral insulin resistance and the inadequate secretion of insulin result in chronic hyperglycemia and various complications [53]. Numerous studies suggest that aging is a risk factor for insulin resistance, but the detailed mechanism of aging effects on the development of dysfunctional β-cells is not clear yet. Unlike type 1 diabetes, in which an early loss of β-cells occurs because of autoimmunity against them, the β-cells in type 2 diabetes undergo initial β-cell compensation and subsequent β-cell dysfunction before many of the β-cells are eventually lost [54]. Compensatory processes include the expansion of the mass of β-cells, enhanced insulin biosynthesis, and increased insulin secretion in response to nutrients [54]. The mass and proliferation of pancreatic β-cells in the presence of obesity and aging conditions are initially elevated by the increased demand for insulin due to the development of insulin resistance [55,56,57,58,59]. However, the eventual failure of β-cells to compensate follows, resulting in insufficient levels of insulin and the development of type 2 diabetes [60,61,62,63].

Ribonucleic acid (RNA) sequencing analyses from mouse islets revealed that SA-β-gal-positive senescent β-cells exhibit downregulated β-cell identity genes (e.g., *Ins1, Pdx1, Mafa, Neurod1*), whereas upregulated genes are involved in the SASP (e.g., *Ccl2, Il1a, Il6, Tnf*) and senescence (e.g., *Cdkn2a*, *Cdkn1a*) compared to SA-β-gal-negative β-cells [53,64]. Additionally, the decreased ability to secrete insulin along with the accompanying downregulation of the expression of *Pdx1, Slc2a2, Ins2,* and *Pcsk1* (prohormone convertase 1/3) were observed in islets from rats and mice aged 22–24 months compared to those aged 2 months [6]. Other recent studies in both humans and rodents also reported that adaptive β-cell replication decreased with age [43,65,66,67]. Furthermore, glucagon-like peptide 1 (GLP-1)-stimulated β-cell proliferation and the compensatory replication of β-cells after low-dose STZ treatment decreased with the increase in participants’ age [65,68]. Consistent with the reduction of β-cell replication, a higher expression of cell-cycle inhibitors, such as *Cdkn1a, Cdkn1b, Cdkn2a, Cdkn2b, and Rb1*, were observed in the islets of mice aged between 22 and 24 months [65].

However, the role of senescence in insulin secretion is still unclear. Several human studies have documented a decline in the secretion of insulin as a result of the aging process [69,70,71,72], whereas certain studies have found a progressive increase in the secretion of basal insulin in elderly people [73]. Additionally, islet transplantation, which allows islets’ autonomous function without systematic metabolic influence from the donor to be studied, demonstrated that the insulin secretion index was significantly higher in islets from younger donors than in islets from older donors [7]. However, nondiabetic and 20-month-old mice that were considered to be elderly exhibited increases in the size of islets and GSIS, concomitant with decreased insulin sensitivity [74]. Another study showed that 10-month-old rats exhibited no change in the secretion of basal or stimulated insulin even with the loss of their β-cell proliferation in response to mitotic stimuli [75].

Although there is little mechanistic understanding of the secretion of insulin during the process of aging, a recent paper documented that β-cell senescence that was induced by an increased p16^Ink4a^ action promoted the secretion of insulin and tolerance for glucose despite the lack of β-cell proliferation. This is suggested to be mediated by an elevation in mTOR and PPAR γ activity and the accompanying enhanced mitochondrial activity [76].

Recent research has reported that senescent β-cells affect the progression of diabetes. In rodent studies, the accumulation of dysfunctional senescent β-cells contributes to an impairment in the tolerance for glucose and the development of diabetes. On the other hand, the removal of the p16^Ink4a^-expressing senescent cells either genetically (using the INK-ATTAC model) or pharmacologically (ABT-263, a Bcl-2 inhibitor) restored glucose tolerance and insulin sensitivity in the type 2 diabetes model [64]. In addition, in the type 1 diabetes model, senescent β-cells contribute to the immune-mediated destruction process, and the removal of senescent β-cells through treatment with senolytic drugs (such as ABT-199 (Bcl-2 inhibitor) and ABT-737 (Bcl-2, Bcl-xL, Bcl-w inhibitor)), prevents the progression of diabetes in the NOD type 1 diabetes mouse model [77].

## 3. Cellular Senescence

Cellular senescence is a necessary biological process frequently seen in the healing of wounds. This provides a protective action against cellular stresses and tumorigenesis [78,79,80]. However, the accumulation of senescent cells with the progression of age contributes to the development of various aging-associated diseases [1,81]. Cellular senescence was first identified in 1961 by Moorhead and Hayflick, who observed that fibroblasts had lost their proliferative capacity after a prolonged culture period (irreversible cell-cycle arrest) [82]. Since then, an increasing number of studies have documented a variety of senescent phenotypes that include irreversible cell-cycle arrest and other phenotypes, such as resistance to cell death, elevated expression and secretion of the senescence-associated secretory phenotype (SASP), morphological changes (enlargement, flattening, and irregular shape), and increased senescence-associated-β-galactosidase (SA-β-gal) activity. The phenotypic outcomes of senescence are diverse, depending on the types of cells, extracellular and intracellular stimuli, and signaling responses. This causes significant difficulties when determining and studying cellular senescence [83]. Currently, several senescence markers and their roles in aging and aging-associated diseases have been documented [83] (Table 1).

### 3.1. Representative Biomarkers That Distinguish Senescent Cells (Cellular Senescent Markers)

Cellular senescence is typically accompanied by several major morphological changes, including flattened, expanded, and vacuolized cell shapes and occasionally numerous or enlarged nuclei. Vacuolation has been linked to an uncontrolled activation of the unfolded protein response (UPR) as well [94].

In addition to morphological characteristics, cellular senescence is being identified using various molecular markers that can distinguish between senescent cells and quiescent or differentiated cells. Several studies have revealed that the p53-p21^Cip1^ and p16^Ink4a^-Rb pathways are frequently activated in senescent cells from an irreversible cell-cycle arrest [86]. The p53-p21^Cip1^ and p16^Ink4a^-Rb pathways are initially identified as tumor suppressor pathways, and p21^Cip1^ and p16^Ink4a^ function as cyclin-dependent kinase (CDK) inhibitors, which block cell cycle progression. To study senescence, the development of various genetic animal models of the p16^Ink4a^ gene, in particular, provides a significantly helpful foundation for detecting and separating senescent cells and studying the effect of senolysis [95]. However, p16^Ink4a^ has reportedly been expressed at a high level in some tumor cells, induced by an oncogenic virus targeting the Rb protein, which limits its use as a sole marker to define senescence [96].

Besides p21^Cip1^ and p16^Ink4a^, SA-β-gal activity is being used extensively as a marker of senescence. SA-β-gal activity is caused by an increased quantity of lysosomal enzymes, which is probably linked to the increased lysosomal biogenesis that can be seen in senescent cells [90]. As in p21^Cip1^ and p16^Ink4a^, SA-β-gal activity is also not enough to be the sole senescent state marker. SA-β-gal activity is detected in non-senescent hair follicles, sebaceous glands, eccrine glands, and ductal cells [97]. Conversely, fibroblasts from human patients with autosomal recessive G_M1_-gangliosidosis lacking lysosomal β-galactosidase activity still undergo cellular senescence [98]. Moreover, requiring freshly prepared or carefully cryopreserved tissue samples for the purpose of measuring SA-β-gal activity limits its efficacy for use in in vivo studies [98]. However, SA-β-gal activity is still used widely as a senescent marker. Additionally, lipofuscin accumulation, senescence-associated heterochromatin foci, and senescence-associated distension of satellites are used as senescent markers [83].

### 3.2. Antiapoptotic Processes in Cellular Senescence and Senolysis

Apoptosis, also known as programmed cell death, employs a complicated and energy-dependent cascade of molecular reactions for the purpose of inducing cell death [99]. The apoptotic process is initiated by either extrinsic or intrinsic death signals. The extrinsic apoptosis pathway (also referred to as a death receptor pathway) is mediated by cell surface death receptors such as the TNF receptor and Fas (also known as CD95 or APO-1) [100]. The intrinsic apoptosis pathway is triggered by intracellular stressors, such as DNA damage, hypoxia, oxidative stress, and ER stress. Both pathways induce the stimulation of the mitochondrial membrane and the release of cytochrome C and other apoptogenic factors from the mitochondrial intermembranous region [101].

Senescent cells have been documented to be not only non-proliferative but also resistant to apoptosis by activating several pro-survival pathways. Bcl-2/Bcl-xL, PI3K/AKT, p53/p21/serpines, dependence receptors/tyrosine kinases, and HIF-1 are upregulated in senescent cells as antiapoptotic pathways [102]. Senolytic drugs, such as dasatinib (dependence receptor inhibitor), quercetin (PI3K inhibitor), and navitoclax (Bcl-2/Bcl-xL inhibitor), suppress antiapoptosis and conversely promote apoptosis responses in senescent cells (Table 2). When senolytic drugs were administered to senescent mouse models, their exercise ability and cardiovascular function improved, and various aging-associated phenotypes, such as impaired hematopoietic stem cell functions and osteoporosis, were ameliorated [95,102].

### 3.3. Senescence-Associated Secretory Phenotype (SASP) and Senomorphic Drugs

Senescent cells can affect neighboring cells with a SASP. Senescent cells secrete many signaling factors (interleukins (ILs), chemokines, and growth factors), proteases, and protein/extracellular matrix (ECM) components. The SASP production and secretion have been documented to be regulated by several transcription factors and chromatin regulators. Although the composition of the SASP varies depending on the types of cells and their cellular stressors, key SASP programs comprising pro-inflammatory IL-6, CXC chemokine ligand 8 (CXCL8, also referred to as IL-8), and monocyte chemoattractant protein-1 (MCP1, also referred to as CCL2) appear in most senescent cells. The SASP has both positive and negative functions in senescent cells. One of the positive functions of the SASP is to activate the immune system to get rid of senescent cells. It is involved in both adaptive and innate immune cell activation and recruitment. However, when these SASPs continuously stimulate chronic inflammation and affect the surrounding cells, adverse aging-associated diseases, such as cancer, arteriosclerosis, and degenerative arthritis, inevitably follow [83,93,126].

Senomorphic agents have the ability to attenuate the key attributes of cellular senescence without inducing cellular death, primarily by suppressing the production and secretion of the SASP. As a result, senomorphics alleviated the senescent phenotypes by returning them to a healthy state or delaying the process of senescence [127]. Senomorphics include free radical scavengers, rapamycin, and inhibitors of IκB kinase (IKK), NF-κB p65, and Janus kinase (JAK) [128] (Table 2). As a famous example of potential antiaging agents, rapamycin (sirolimus), which is a well-known mTOR inhibitor, has been shown to extend the lifespan of various animal models, including nematodes, fruit flies, and mice [129,130,131]. Rapamycin inhibits IL-2 and other cytokine receptor–dependent signal transduction processes from occurring, resulting in significant immunosuppressive and antiproliferative effects. These properties are derived from the capacity to inhibit mTOR, a conserved serine/threonine kinase that regulates cell growth and death in response to the host’s nutritional status, growth factors, and stress signals [132]. Metformin is a well-known pharmacological drug used to treat type 2 diabetes. It has been shown to alleviate various aging-associated disorders, such as impaired glucose homeostasis and chronic inflammation involving the pathways of AMPK/SIRT1, ILs, mTOR, and IKK/NF-κB [123,124,125].

## 4. ER Stress, UPR, and Aging-Associated β-Cell Dysfunction

### 4.1. ER Stress and UPR in β-Cells

**Figure 2:**
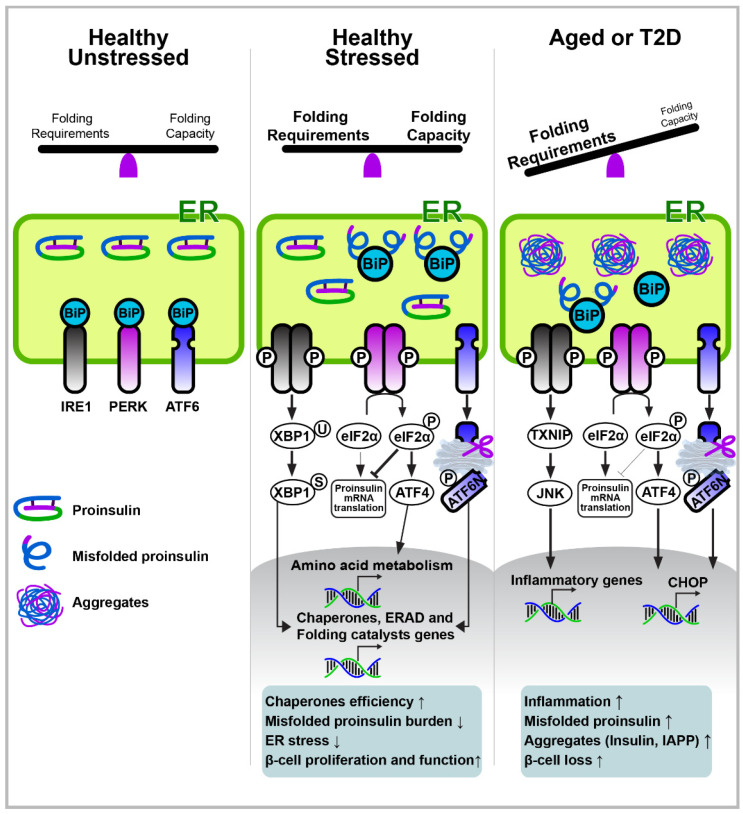
Pancreatic β-cell ER stress and the UPR pathway in aging and type 2 diabetes. In the absence of stress, IRE1, ATF6, and PERK exist in combination with the ER chaperone BiP/GRP78. Under ER stress conditions, BiP is released from IRE1, ATF6, and PERK, resulting in UPR initiation. The adaptive UPR outputs maintain cellular homeostasis by increasing molecular chaperones for protein folding, increasing ERAD, and reducing the translation of mRNA. However, chronic stress, such as aging or type 2 diabetes, upregulates UPR dysfunction in β-cells to induce inflammation, proliferation loss, and apoptosis. Moreover, the increased supply of unfolded monomers (i.e., insulin, IAPP) exceeds their disposal capacity, leading to the production of misfolded protein aggregates.

The ER is an intracellular organelle where cellular lipids are produced, cellular calcium is stored, and most secretory and transmembrane proteins are translated and formed into their native structure [3,133]. The maintenance of ER homeostasis is critical because impaired ER homeostasis (ER stress) has been linked to various aging-associated diseases, such as cardiovascular diseases, type 2 diabetes, obesity, and neurodegenerative diseases [3,133]. Cells employ sophisticated signaling responses, known as the UPR, as a result of ER stress. The UPR senses an imbalance in the protein homeostasis in cells and tries to restore balance and homeostasis in the ER. The typical number of pancreatic β-cells produced is approximately 6000 proinsulin molecules per second. Thus, in the prediabetic state, β-cells must adapt their ER machinery to a hyperglycemic environment to promote the production of insulin. To manage this burden, β-cells increase their ER size, and the activation of the UPR plays a central role in the adaptation of β-cells and their subsequent functions under a hyperglycemic condition in type 2 diabetes (Figure 2).

The UPR is initiated by three transmembrane proteins, including the protein kinase PKR-like ER kinase (PERK), inositol-requiring enzyme 1 (IRE1), and activating transcription factor 6 (ATF6). In the absence of stress, IRE1, ATF6, and PERK exist as inactive monomers in combination with the ER chaperone BiP/GRP78 (Figure 2). Under ER stress conditions, BiP is released from IRE1, ATF6, and PERK, resulting in the initiation of UPR. The UPR attenuates protein translation via PERK-mediated eIF2α phosphorylation to reduce the influx of protein to the ER, and it simultaneously induces the expression of chaperones that increase the folding capacity via ATF6 and IRE1α–XBP1. The UPR also removes misfolded and unfolded proteins through ER-associated degradation (ERAD) and autophagy. However, if an overload of unfolded or misfolded proteins in the ER is not resolved, the UPR eventually induces apoptosis via the activation of the C/EBP homologous protein (CHOP), which is a downstream signaling factor of the PERK–ATF4 pathway.

IRE1α contains serine/threonine kinase and endoribonuclease (RNase) domains and is activated via oligomerization and trans-autophosphorylation after being released from BiP during ER stress. The activated IRE1α triggers the splicing of XBP1 mRNA (XBP1s; active form) via its RNase activity. The IRE1α–XBP1 signaling pathway eventually facilitates the transcription of various genes, including ER chaperones, folding catalysts, and ERAD machineries (Figure 2). Mice with XBP1-deletion in β-cells display defects in proinsulin processing, insulin secretion, and adaptive β-cell proliferation, leading to glucose intolerance and hyperglycemia [134,135]. These phenotypes result from the combined lack of XBP1s activity and the compensatory hyperactivation of IRE1α. The deficiency of XBP1s fails to induce a sufficient expression of ER chaperones required for the process of proinsulin folding and processing, whereas the hyperactivation of IRE1α leads to the downregulation of a subset of mRNAs, including insulin and proinsulin-processing enzymes [134,136,137]. In contrast, the sustained production of XBP1s in rat islet cells leads to impaired GSIS and elevated β-cell apoptosis, with a reduced *Pdx1* and *Mafa* expression [138]. Additionally, IRE1α deletion in β-cells results in hypoinsulinemia and hyperglycemia due to a decrease in insulin production and secretion, probably from a lack of functional XBP1s [139,140].

ATF6 is a type II transmembrane protein with a basic leucine zipper (bZIP) domain, and its retention in the ER suppresses its transcriptional activity. However, ER stress and BiP release from ATF6 allow ATF6 to move to the Golgi, where ATF6 is cleaved by two proteases (S1P and S2P), and its bZIP domain is released and transported into the nucleus. ATF6 promotes the transcriptional expression of ER chaperones and other ER homeostatic factors to help with the restoration of ER homeostasis like XBP1s. Therefore, ATF6 expression under β-cell ER stress may help insulin folding and processing to occur [141,142]. Furthermore, the initial insulin demand and ER-stress-mediated UPR activation were reported to promote β-cell proliferation via the activation of ATF6 [142] (Figure 2).

PERK is activated by ER stress from homodimerization and autophosphorylation after unbinding from BiP. Activated PERK further phosphorylates eIF2α to attenuate protein translation [143] (Figure 2). Paradoxically, the translations of certain proteins, such as ATF4 and ATF5, are upregulated under PERK activation due to the upstream open reading frames in the 5′ untranslated region of their mRNAs. ATF4 transcriptionally promotes the expression of *TRIB3, DDIT3* (CHOP), *EIF4EBP1* (4E-BP1), *PPP1R15A* (GADD34), and other genes that are involved in the metabolization of amino acids [3,133]. Among ATF4 targets, CHOP is well documented as an eventual inducer of ER-stress-mediated cell death [3,144]. In addition to ER stress, eIF2α phosphorylation and subsequent ATF4 translation are induced by other cellular stimuli, such as a heme deficiency, mitochondrial stress, amino acid deprivation, and viral infection via other kinases (GCN2, PKR, and HRI). Hence, the ATF4 pathway is referred to as the integrated stress response [145,146]. Numerous in vivo human and rodent studies have demonstrated the crucial role of the PERK–ATF4 pathway in β-cell biology and pathology [147,148,149,150,151,152,153,154,155]. For example, mouse models and human genetics studies found that a deficiency in PERK in β-cells triggers ER stress, β-cell loss, and severe hyperglycemia [148,153,154,155,156]. Additionally, preventing the PERK-mediated phosphorylation of eIFα leads to glucose intolerance in mice [147,150]. ATF4 deletion in Akita mice results in more severe hyperglycemia [157]. These observations suggest that the completely depleted PERK–ATF4 pathway creates a condition of deterioration in β-cells. However, increased ER stress has been suggested to be a contributing factor to the impaired functioning of β-cells during the progression of type 2 diabetes. Additionally, rather than depleting PERK activity, decreasing it paradoxically promotes GSIS [156,158,159], and the deletion of the PERK–ATF4 pathway’s downstream signaling factors, such as CHOP, TRIB3, or 4E-BP1 in β-cells, alleviates ER stress and prevents the loss of β-cells [149,151,152,160,161,162]. Elevated ER stress in β-cells triggers the activation of IRE1α and PERK, eventually leading to the death of β-cells via the induction of the thioredoxin-interacting protein and subsequent inflammasome activation [163,164].

Besides the examples mentioned above, other reports document the crucial roles of ER stress–related factors in β-cell biology, as in studies with WFS1 [165,166], ERO1β [167,168], p58^IPK^ [169], ERp5 (PDIA6) [170], GRP94 [171], and ERAD machinery (Sel1–Hrd1) [172].

### 4.2. ER Stress and Its Crosstalk with Other Cellular Stresses

In addition to ER stress, other cellular stresses, such as mitochondria dysfunction and oxidative stress, have been found to play a critical role in aging and aging-associated illnesses [133]. Oxidative stress is frequently caused by the overwhelming production of the reactive oxygen and nitrogen species and has been suggested to induce senescent phenotypes. However, the exact molecular mechanism of oxidative stress in cellular senescence is not understood very well [173]. Reactive oxygen species (ROS) are generated primarily from the mitochondria and have been shown to induce oxidative damage in macromolecules, such as lipids, nucleic acids, and proteins. Mitochondria are double membrane–bound organelles that generate ATP through oxidative phosphorylation (OXPHOS) in eukaryotic cells. Mitochondrial dysfunction is also considered to be a typical hallmark of aging [174]. Several studies have frequently observed the impairment of mitochondrial biogenesis, decreased mitophagy, and hyperfused mitochondrial networks in senescent cells [175]. These were linked to deteriorated OXPHOS and increased ROS production [176,177,178]. The mitochondrial production of ROS occurs as a by-product of electrons that leak along the electron transport chain during respiration.

Interestingly, many cellular stresses influence each other. ER stress inducers are often found to trigger other cellular stresses, such as inflammation, oxidative stress, and mitochondrial stress [133]. In particular, ER’s physical interaction with the mitochondria has reportedly been found to contribute to the ER’s and the mitochondria’s biological functions in a crucial way [179]. The tubular ER forms membrane contact sites (MCSs) with other organelles including mitochondria [180]. The ER-mitochondria contact sites, also called mitochondrial-associated membranes (MAMs), have demonstrated a critical role in the process of mitochondrial fusion and fission, lipid synthesis, and the transfer of calcium from the ER to the mitochondria [180]. Many ER and mitochondrial proteins have been identified in MAM, including MFN and DRP1 (mitochondrial fusion and fission), ORP5/8 (lipid synthesis and transport between the ER and mitochondria), and VDAC and IP3R (calcium transport) [180,181]. ER stress in β-cells (MIN6) induced via thapsigargin resulted in perturbations of the mitochondrial membrane’s potential and apoptosis, which was attenuated by blocking the ER calcium channels IP3R and RyR [182]. Moreover, the ER UPR factor ATF4 crucially mediates the mitochondrial stress response [183].

### 4.3. Proteostasis Imbalance in β-Cells—The Risk of Proinsulin Accumulation and Aggregation

Protein homeostasis (proteostasis) refers to the proper control of proteins’ structure, interactions, and levels of expression. Proteostasis is maintained by various regulatory networks, including molecular chaperones and proteolytic systems [184]. Impaired proteostasis contributes to numerous aging-associated diseases, as famously exemplified in several neurodegenerative diseases, such as Alzheimer’s disease and Parkinson’s disease [2]. Similar pathologies to neurodegenerative diseases that are caused by impaired proteostasis and the accumulation of misfolded proteins or their aggregates have also been observed in β-cells.

According to previous studies, approximately 10–20% of circulating immunoreactive insulin is proinsulin, which is cleared from the plasma slower than mature insulin [185,186]—in the elderly, circulating proinsulin concentrations increase, as does hyperglycemia, which is related to the aging process [187,188]. In both diabetic and nondiabetic individuals, the proinsulin-to-insulin ratio (PI/I) was negatively correlated with the secretion of insulin [189,190]. An increased level of proinsulin or the PI/I ratio has also been suggested to be an indicator of β-cell dysfunction and type 2 diabetes [186,191,192,193,194,195]. Impaired ER homeostasis, caused by lipotoxicity, inflammation, hyperglycemia, and aging, has been found to induce the misfolding of proinsulin [196] (Figure 2). This has been suggested to induce the formation of insulin aggregates and β-cell failure, as exemplified in Akita mice and several human genetic studies [20,30,31].

### 4.4. Proteostasis Imbalance in β-Cells—Islet Amyloid Polypeptide Aggregation

Islet amyloid polypeptide (IAPP or Amylin) is a length of a 37-amino acid residue peptide that is simultaneously expressed and secreted with insulin from pancreatic β-cells. Initially, IAPP is expressed as an 89-amino acid pre-pro-peptide that is subsequently processed in the ER and the Golgi to become a mature IAPP. Additionally, the amidation of the C-terminal glycine residue of IAPP is observed similar to other hormones and is expected to be essential for its potent biological activity, although this is not completely understood yet. The IAPP of most mammalian species, including humans (hIAPP), is prone to forming fibrils with its β-sheet areas, resulting in the conversion to the amyloid-like aggregation. In contrast, rodent IAPP (rIAPP) does not form amyloidogenic structures because of the presence of proline residues at the IAPP20–29 region, which disrupt the formation of a β-sheet structure. Thus, β-cell-specific hIAPP-transgenic mice and rats are frequently used to study the pathophysiology of IAPP aggregation. These rodent models display amyloid deposits of IAPPs and the development of impaired insulin secretion, β-cell loss, and diabetes [197,198,199]. β-Cell IAPP amyloid deposition has been linked to oxidative stress, ER stress, inflammation, damaged β-cell membranes, and the eventual apoptosis of β-cells [198,200,201] (Figure 2). In addition to phenotypic studies using hIAPP animal models, an increased deposition of the islet amyloid has been reported in human subjects with type 2 diabetes [202]. Furthermore, among patients with type 2 diabetes, more amyloid deposition was found in the islets of the elderly group (>85 years) than in the middle-aged group (45 to 87 years) [203]. Relieving ER stress alleviates pathologies in islet amyloid-associated β-cells. The administration of 4-phenylbutyrate (PBA), a chemical chaperone that has been known to alleviate ER stress, alleviated amyloid deposition, glucose intolerance, and hyperglycemia in hIAPP-transgenic mice [204]. In addition, the attenuation of autophagy, which has been found to reduce with age and in type 2 diabetes [205,206], further deteriorates the dysfunction of islet amyloid-associated β-cells, including the loss of β-cells and the development of hyperglycemia [207,208].

## 5. Future Perspectives—Targeting ER Stress and Senescent β-Cells as Probable Therapeutics for Aging-Associated Type 2 Diabetes

Various perturbations in homeostasis that frequently curtail the proper functioning of the ER accompany aging and lead to numerous aging-associated diseases, including neurodegenerative diseases, obesity, and type 2 diabetes. Both developed and developing countries have an aging society, and many aging-associated diseases increase rapidly to become an enormous social and economic burden. Pancreatic β-cells are a major organ used to control the homeostasis of glucose, and their dysfunction determines the severity of both type 1 and type 2 diabetes. Accordingly, numerous medications have been developed to target the pancreatic β-cells to ameliorate hyperglycemia and other diabetic complications (Table 3). However, these β-cell-targeting therapies only enhance the remaining β-cells’ function instead of alleviating and reversing aging- and type 2 diabetes-associated β-cell dysfunction. Thus, a mechanistic understanding of β-cell malfunction and the development of therapeutics to address it would benefit people who have type 2 diabetes.

Recent studies have discovered that β-cell ER stress and senescence have been found to be critically involved in β-cell pathologies in type 2 diabetes and aging. The senolytic mouse model (INK-ATTAC) or senolytic reagent (ABT-263) used to remove p16-positive senescent β-cells have demonstrated an improvement in the functioning of β-cells, alleviated β-cell senescence and SASP production, and glucose homeostasis under insulin-resistant conditions [64]. Additionally, numerous studies have identified general ER stress modulators, such as 4-PBA, taurine-conjugated ursodeoxycholic acid (TUDCA), celastrol, and withaferin A [133], and the administration of 4-PBA and TUDCA has reportedly improved the functioning of β-cells, including the secretion of insulin, during in vitro and in vivo studies [209,210,211]. Furthermore, various chemicals that specifically target UPR factors, including IRE1α, PERK, eIF2B, GADD34, and ATF6, have been identified, and some of them (especially targeting the PERK-associated pathway) have been shown to alleviate neurological diseases linked to aging and the aggregation of protein [212]. Indeed, PERK inhibitors have been shown to improve the secretion of insulin [158,159]. Additionally, the targeting of β-cell-specific UPR factors has been demonstrated successfully using the GLP-1-linked antisense oligonucleotides [152]. Although it has not yet been fully explored, targeting ER stress is one way to alleviate aging-associated β-cell pathologies.

## Figures and Tables

**Table 1:** Representative senescence phenotypic traits and related biomarkers.

Senescent Phenotypes	Senescent Markers	Reference
Morphological changes	Flattened, expanded, and vacuolized cell shape Numerous or enlarged nuclei	[84,85]
Cell cycle arrest	Elevated p53-p21^Cip1^ and p16^Ink4a^-Rb activities	[86,87,88,89]
Lysosomal expansion	Increased lysosomal mass and activities (SA-β-gal and lipofuscin)	[83,90,91]
Chromatin reorganization	Elevated senescence-associated heterochromatin foci (SAHF) and senescence-associated distension of satellites (SADS)	[83,92]
Senescence-associated secretory phenotype (SASP)	Increased production and secretion of the SASP components (interleukins, chemokines, and growth factors)	[83,92,93]

**Table 2:** Senotherapeutics drugs and their targets and effects on senescent β-cell or other metabolic disease-related pathologies.

Targets	Drug	Type	Effects on Aging Model	Ref
Bcl-2 family inhibitors	ABT-199 (Venetoclax)	Senolytic	Preserving β-cell mass and preventing T1D pathologies	[77,103]
	ABT-737	Senolytic	Decreasing Cdkn2a- and MMP2-expressing β-cells	[64,77,104]
	Navitoclax (ABT-263)	Senolytic	Improving hematopoietic parameters Improving glucose metabolism and β-cell function	[64,103,105,106]
Src/tyrosine kinase inhibitor	Dasatinib	Senolytic	Decreasing senescent cell numbers and restoring β-cell identity in T2D animal	[107,108]
Inhibition of PI3-kinase activity	Quercetin	Senolytic	Reducing senescence-associated features in senescent adipocytes Decreasing senescent cell numbers and restoring β-cell identity in T2D animal	[107,109]
Up-regulating SIRT1	Luteolin	Senolytic	Beneficial effects on patients with age-related macular degeneration	[110,111]
Foxo4/p53 interfering peptide	Foxo4-DRI (Proxofim)	Senolytic	Restoring fitness, fur density, and renal function in aging mice	[112]
mTOR inhibitor	Rapamycin	Senomorphic	Extending life span in various animal models	[113,114,115]
JAK inhibitor	Ruxolitinib	Senomorphic	Preventing progerin-induced senescence Alleviating inflammation and enhancing physical function in aged mice	[116,117]
Multi-pathway	Fisetin	Senolytic Senomorphic	Extending health span and lifespan	[118,119]
	Resveratrol	Senomorphic	Ameliorating β-cell senescence and loss in senescent animal model	[120]
	Curcumin	Senolytic	Preventing vascular aging	[121,122]
	Metformin	Senomorphic	Alleviating age-related disorders and improving health span	[123,124,125]

**Table 3:** Pancreatic β-cell-targeting antihyperglycemic agents for patients with type 2 diabetes.

Class	Senescent Markers	Mechanism	Side Effects
Sulfonylureas	Tolbutamide Glimepiride Glipizide Glyburide	Interacting with the sulfonylurea receptors Insulin secretagogues	Weight gain Hypoglycemia
Meglitinide	Repaglinide Nateglinide, Mitiglinide	Interacting with the sulfonylurea receptors Insulin secretagogues	Weight gain Hypoglycemia
DPP4 inhibitors	Alogliptin Anagliptin Sitagliptin Saxagliptin Linagliptin Omarigliptin Teneligliptin Vildagliptin	Increasing the activity of GLP-1 Potentiating insulin secretion	GI disturbance Pancreatitis
GLP-1 analogs	Exenatide Liraglutide Albiglutide Dulaglutide	Prolonging the half-life of GLP-1 Increasing insulin secretion and synthesis Inhibiting glucagon release	GI disturbance Pancreatitis
